# Alcohol‐attributed disease burden in four Nordic countries between 2000 and 2017: Are the gender gaps narrowing? A comparison using the Global Burden of Disease, Injury and Risk Factor 2017 study

**DOI:** 10.1111/dar.13217

**Published:** 2020-11-18

**Authors:** Emilie E. Agardh, Peter Allebeck, Pär Flodin, Peter Wennberg, Mats Ramstedt, Ann Kristin Knudsen, Simon Øverland, Jonas Minet Kinge, Mette C. Tollånes, Terje A. Eikemo, Jens Christoffer Skogen, Pia Mäkelä, Mika Gissler, Knud Juel, Kim Moesgaard Iburg, John J. McGrath, Mohsen Naghavi, Stein Emil Vollset, Emmanuela Gakidou, Anna‐Karin Danielsson

**Affiliations:** ^1^ Department of Global Public Health Karolinska Institutet Stockholm Sweden; ^2^ Department of Public Health Sciences Stockholm University Stockholm Sweden; ^3^ Swedish Council for Information on Alcohol and Drugs Stockholm Sweden; ^4^ Department of Clinical Neurosciences Karolinska Institutet Stockholm Sweden; ^5^ Centre for Disease Burden The Norwegian Institute of Public Health Bergen Norway; ^6^ Department of Psychosocial Science University of Bergen Bergen Norway; ^7^ Centre for Fertility and Health Norwegian Institute of Public Health Oslo Norway; ^8^ Department of Health Management and Health Economics University of Oslo Oslo Norway; ^9^ Norwegian Organization for Quality Improvement of Laboratory Examinations Haraldsplass Deaconess Hospital Bergen Norway; ^10^ Centre for Global Health Inequalities Research, Department of Sociology and Political Science Norwegian University of Science and Technology Trondheim Norway; ^11^ Department of Health Promotion Norwegian Institute of Public Health Bergen Norway; ^12^ Department of Public Health, Faculty of Health Sciences University of Stavanger Stavanger Norway; ^13^ Alcohol and Drug Research Western Norway Stavanger University Hospital Stavanger Norway; ^14^ Alcohol and Drugs Unit Finnish Institute for Health and Welfare Helsinkim Finland; ^15^ Information Services Department Finnish Institute for Health and Welfare Helsinki Finland; ^16^ Department of Neurobiology, Care Sciences and Society Karolinska Institutet Stockholm Sweden; ^17^ National Institute of Public Health University of Southern Denmark Copenhagen Denmark; ^18^ Institute of Public Health Aarhus University Aarhus Denmark; ^19^ National Center for Register‐based Research, Business and Social Sciences Aarhus University Aarhus Denmark; ^20^ Queensland Brain Institute The University of Queensland Brisbane Australia; ^21^ Queensland Centre for Mental Health Research The Park Centre for Mental Health Brisbane Australia; ^22^ Institute for Health Metrics and Evaluation University of Washington Seattle USA

**Keywords:** alcohol, disease burden, Nordic countries, global burden of disease

## Abstract

**Introduction and Aims:**

The gender difference in alcohol use seems to have narrowed in the Nordic countries, but it is not clear to what extent this may have affected differences in levels of harm. We compared gender differences in all‐cause and cause‐specific alcohol‐attributed disease burden, as measured by disability‐adjusted life‐years (DALY), in four Nordic countries in 2000–2017, to find out if gender gaps in DALYs had narrowed.

**Design and Methods:**

Alcohol‐attributed disease burden by DALYs per 100 000 population with 95% uncertainty intervals were extracted from the Global Burden of Disease database.

**Results:**

In 2017, all‐cause DALYs in males varied between 2531 in Finland and 976 in Norway, and in females between 620 in Denmark and 270 in Norway. Finland had the largest gender differences and Norway the smallest, closely followed by Sweden. During 2000–2017, absolute gender differences in all‐cause DALYs declined by 31% in Denmark, 26% in Finland, 19% in Sweden and 18% in Norway. In Finland, this was driven by a larger relative decline in males than females; in Norway, it was due to increased burden in females. In Denmark, the burden in females declined slightly more than in males, in relative terms, while in Sweden the relative decline was similar in males and females.

**Discussion and Conclusions:**

The gender gaps in harm narrowed to a different extent in the Nordic countries, with the differences driven by different conditions. Findings are informative about how inequality, policy and sociocultural differences affect levels of harm by gender.

## Introduction

Alcohol use is the seventh leading risk factor for death and disability globally, resulting in more than 100 million healthy years of life lost. In 2017, alcohol use accounted for 7% of the total disease burden in males and 1.3% in females [1]. This reflects the fact that males consume more alcohol than females and, consequently, suffer more from alcohol‐related disease and death [2]. It is known that the size of the gender gap in alcohol use varies between countries [3], but it is less clear how differences between genders in levels of all‐cause and cause‐specific harm have been affected, and how the differences have developed over time, which may have implication for effective policy making. The largest differences in alcohol use between males and females are observed in low‐ and middle‐income countries, and the smallest differences are found in high‐income countries like the Nordic countries [2, 4]. It has been suggested that male to female differences are smaller in countries with greater gender equality, such as the Nordic countries [5, 6, 7]. During the past decades, there have been signs of gender convergence, reflecting differential increases or decreases in male and female alcohol use, in many countries [8, 9, 10, 11, 12], including the Nordic ones [13, 14, 15]. The convergence is suggested to be primarily attributable to a rise in female drinking rather than a reduction in male drinking [16, 17]. This is hypothesized to partly reflect females adopting male behavioural patterns, reflecting the influence of participation in the labour market, financial independence or mixed‐gender drinking occasions [18, 19]. There is a general concern about this convergence, in part because females may be more vulnerable when affected by alcohol, and also develop medical problems such as alcohol use disorders more quickly than males [3].

Regulation of availability, high taxes and price controls are effective alcohol policies [20]. Although the Nordic countries have strong similarities in terms of population and social and welfare characteristics, there are notable differences in consumption and in the use of alcohol control policies over time [21, 22]. Therefore, there has been a strong interest in following and comparing consumption and alcohol‐related harm in these countries to promote an understanding of the underlying determinants and policies influencing drinking behaviour and adverse health effects. While Sweden and Norway have long had relatively restrictive alcohol policies, Denmark, and more recently Finland, have been more liberal. In a previous study, we showed that the all‐cause variation in levels of alcohol‐attributed disease burden between these countries by and large reflected the differences in alcohol policy [23].

On the other hand, in a more recent study, we showed that the all‐cause alcohol‐attributed disease burden was particularly high among Danes of both genders and Finnish males, and that gender differences were larger in Finland than in Denmark, Sweden or Norway [24]. Since males and females in the same country share the policy context, this implies that other factors are involved, such as socio‐cultural differences related to consumption [25, 26].

Following and comparing the magnitude of gender differences in health burden of alcohol over time is important for public health. Identifying key areas in which most of the harm occurs is crucial to laying a foundation for policy, to promoting an understanding of underlying determinants behind differences, as well as to evaluating the impact of policies. However, existing studies in the Nordic countries have been limited by focusing only on levels of consumption [6, 27, 28], not investigating the patterns of cause‐specific alcohol‐attributed disease burden in males and females separately and over time [23], or investigating different death or disease outcomes [22, 28, 29]. Therefore, this gives only a partial picture of the problem.

The Global Burden of Disease (GBD) study offers a framework for a more complete analysis. The GBD study captures all‐cause and cause‐specific alcohol‐attributed disease burden by combining premature death (years of life lost; YLL) and disability (years lived with disability; YLD) into one single estimate: disability‐adjusted life‐years (DALY). This means that DALYs capture both fatal and non‐fatal health outcomes. A key strength is that the disease burden due to alcohol is estimated systematically and uniformly for males and females by geography and over time [2]. In this study, we focus on gender, seen in a binary way, since these are the data available.

While the GBD study has estimated the burden of alcohol for all countries worldwide [2, 30], these results have not previously been analysed at the cause‐specific level with a focus on gender gaps within the Nordic context. Based on results from the Global Burden of Disease and Injuries and Risk Factors 2017 study [1], we analysed the alcohol‐attributed disease burden by DALYs in males and females in Finland, Denmark, Sweden and Norway between the years 2000 and 2017. More specifically, we aimed to:

1. Assess and compare gender differences in all‐cause and cause‐specific alcohol‐attributed DALYs; and
2. Examine whether the gender gaps in alcohol‐attributed DALYs narrowed or expanded over the study period.

## Methods

### *The global burden of disease study*

The GBD study is currently the leading global information system for tracking and comparing disease burden and contribution of risk factors to disease burden and estimates DALYs as an overall summary measure of population health. The GBD provides estimates for 359 diseases and injuries, 282 causes of death and 84 risk factors, including alcohol use for males and females since 1990. The methods have been described in detail in summary papers and appendices in dedicated issues of *The Lancet* [30]. DALYs measure the gap between an ideal situation where everyone lives a long life in full health, and the current health state of a population, and are calculated by adding together two components: YLLs and YLDs.

YLLs are calculated by multiplying the number of deaths from each cause of death in each age‐group by a reference life expectancy at that age. In the Nordic countries, data on mortality and causes of death are based on vital registration from the cause of death registers.

YLDs take into consideration the prevalence of non‐fatal causes in the population and the health loss associated with each condition, measured by using a disability weight. Disability weights quantify health loss associated with non‐fatal causes and range from zero (perfect health) to one (death). The GBD uses all available health data sources on causes as these have been identified through systematic searches and reviews of both published and unpublished studies. Information on the data input sources is available in the GBD Global Health Data Exchange platform hosted by the Institute for Health Metrics and Evaluation [31]. To generate internally consistent estimates of prevalence, incidence, remission, duration and excess mortality from each non‐fatal health condition, all data are modelled in the Bayesian meta‐regression tool, DisMod‐MR 2.1 (Disease Modeling‐Metaregression). The YLD estimates are derived through this process.

### *Burden of disease attributed to alcohol*

The GBD applies a comparative risk assessment approach in which the observed health outcomes are compared with those that would have been observed with a counterfactual level of exposure that minimises health loss. Calculations are based on three key steps.

1. Estimating the effect of different levels of alcohol use on disease outcomes, that is, relative risks (RR). Alcohol has been causally related to approximately 45 diseases when neoplasms, cardiovascular diseases, injuries, self‐harm and violence, and tuberculosis are broken down into more detailed levels (see Tables S1 and S2, Supporting Information). The RRs in each risk factor‐outcome pair are estimated from systematic reviews and meta‐analyses of published literature, usually giving a continuous risk function over average daily dose in grams of pure alcohol for each outcome. Dose–response curves for RRs have been derived for 23 risk‐outcome pairs using DisMod ODE [2].
2. Estimating the distribution of alcohol consumption in countries by age and for males and females, using data on both alcohol‐stocks and individual‐level alcohol consumption. First, population‐level alcohol stocks in litres per capita are estimated using sales data from the United Nations Food and Agriculture database and the World Health Organization's Global Information System on Alcohol and Health databases. These estimates are adjusted for tourist consumption and unrecorded alcohol stocks [2]. Second, the individual‐level consumption from surveys (i.e. prevalence of current drinkers, level of consumption in litres of pure ethyl alcohol per day and prevalence of abstainers) are modelled for each age by males and females. Third, the age‐ and male and females‐specific patterns of consumption are taken from the individual level data; however, since these have been shown to underestimate consumption [2], they are rescaled to correspond to the level of consumption in the adjusted alcohol‐stock data.
3. Estimating the population attributable burden. The contribution of alcohol to disease burden is estimated by using the distribution of alcohol consumption in the population, the disease‐specific RRs at each level of consumption and the burden of disease for each of the 23 health outcomes in each population. For some outcomes, alcohol has a protective effect at low levels of consumption, and this is taken into account in the final estimation of attributable burden.

### *Analytical strategy*

We compared GBD results for age‐standardised rates of DALYs per 100 000 population with 95% uncertainty interval (UI) for males and females in the Nordic countries at 2‐year intervals between 2000 and 2017. UIs reflect uncertainty from data sources, model specification, stochastic variation and measurement bias. Age‐standardised rates adjust for differences in total population and changes in age‐specific population sizes over time, and allow comparison of alcohol‐attributed health outcomes over time. All estimates were extracted from the GBD Global Health data Exchange platform [31].

We calculated absolute differences (males–females) by all‐cause and cause‐specific alcohol‐attributed disease burden to reflect the gender gap in levels of DALYs over time. To study the gender gap change between the years 2000 and 2017, percent change in absolute differences were calculated as follows: 100*(male–female difference in DALY rates_2017_ − male–female difference in DALY rates_2000_)/(male–female differences in DALY rates_2000_). To get an indication of the size of the differences in all‐cause DALYs, and percentage change in relative differences between the years 2000 and 2017, we also calculated relative gender differences by ratio (male/female) as follows: 1 − (male/female ratio in DALY rates_2017_)/(male/female ratio in DALY rates_2000_).

We present DALY estimates for the following specific causes: alcohol use disorder, self‐harm, interpersonal violence, transport injuries, unintentional injuries, cirrhosis, neoplasms, cardiovascular diseases, diabetes, epilepsy, pancreatitis, lower respiratory infections and tuberculosis. Details on the type of neoplasm and cardiovascular disease are given in Table S1, and type of transport injury or type of self‐harm in Table S2.

## Results

### *The all‐cause and cause‐specific alcohol‐attributed disease burden*

**Figure 1:**
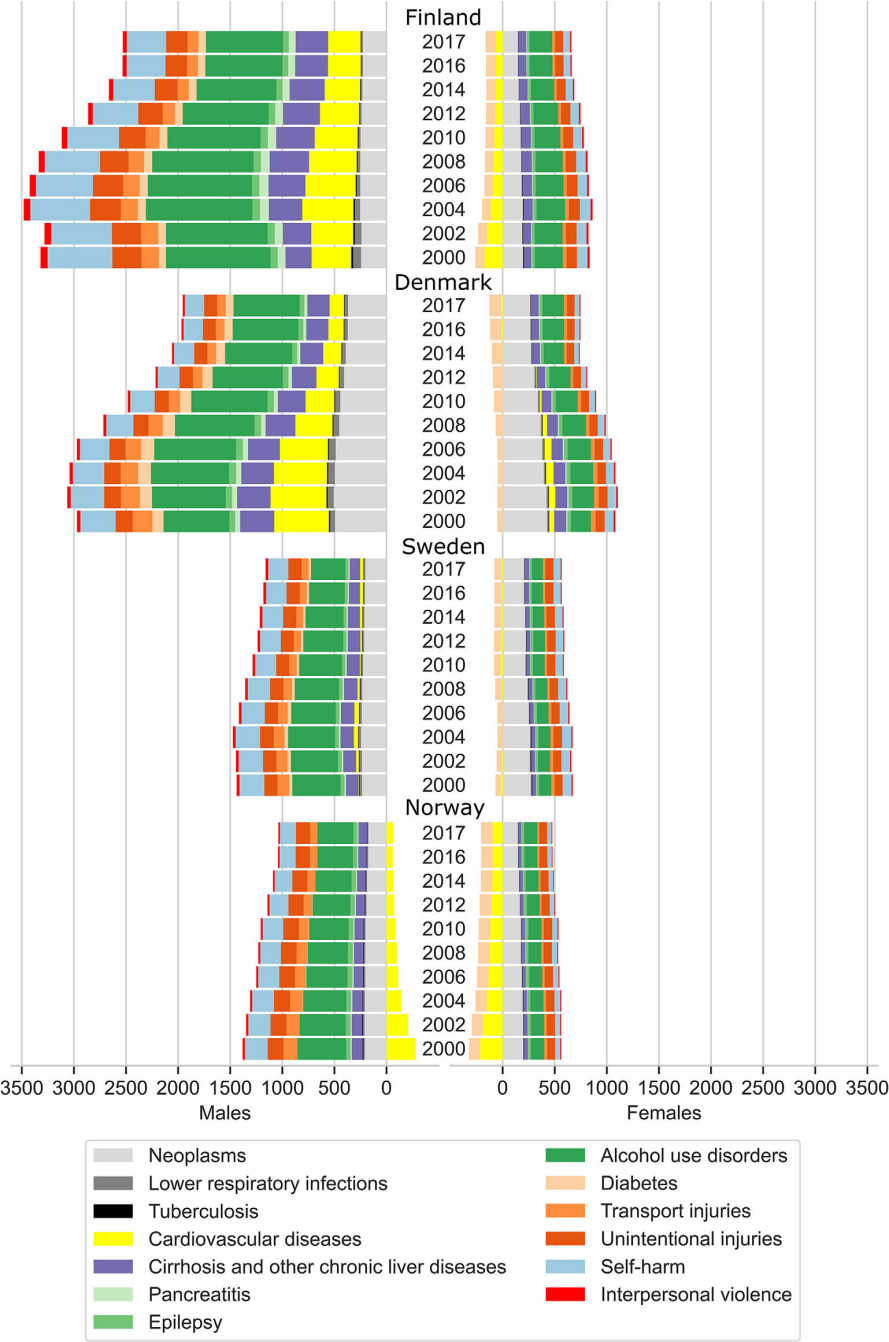
Overall and cause‐specific alcohol‐attributed disease burden by age‐standardised disability‐adjusted life‐years rates per 100 000 by males and females in the Nordic countries between 2000 and 2017.

Finnish males had the highest alcohol‐attributed disease burden during the study period, but with an all‐cause age‐standardised DALYs per 100 000 population that decreased by 24% from 3346 (95% UIs 2709, 4067) in 2000 to 2531 (95% UIs 1983, 3192) in 2017, with a peak in 2004 (Figure 1 and Table 1). In Finnish females, DALYs decreased by 13% from 578 (95% UIs 346, 830) in 2000 to 497 (95% UIs 309, 716) in 2017, with the highest levels in 2004–2010.

**Table 1:** Alcohol‐attributed disease burden in age‐standardised DALY rates per 100 000 by males and females in four Nordic countries 2000 and 2017, and percentage change 2000–2017 by all‐cause and cause‐specific disease burden

	Males			Females		
	Age‐standardised DALYs per 100.000 with 95% uncertainty intervals		Percent change in age‐standardised DALYs per 100.000	Age‐standardised DALYs per 100.000 with 95% uncertainty intervals		Percent change in age‐standardised DALYs per 100.000
*Finland*	2000	2017	2000–2017	2000	2017	2000–2017
All alcohol‐attributed causes	3346 (2709–4067)	2531 (1983–3192)	−24%	578 (346–830)	497 (309–716)	−13%
Alcohol use disorders	1005 (873–1159)	739 (616–844)	−27%	268 (202–327)	217 (169–270)	−19%
Neoplasms	244 (217–272)	227 (191–268)	−7%	194 (164–222)	146 (114–180)	−25%
Cirrhosis and other chronic liver diseases	251 (209–287)	313 (248–373)	25%	70 (56–85)	78 (61–95)	11%
Cardiovascular diseases	380 (−40–775)	213 (33–616)	−18%	−175 (−325‐ ‐19)	−67 (−148–23)	61%
Self‐harm	646 (380–874)	377 (214–534)	−42%	102 (16–177)	66 (11–118)	−35%
Unintentional injuries	280 (94–494)	206 (70–374)	−26%	98 (35–188)	97 (27–156)	−19%
*Denmark*	2000	2017	2000–2017	2000	2017	2000–2017
All alcohol‐attributed causes	2971 (2464–3468)	1956 (1456–2505)	−34%	1030 (748–1334)	620 (358–946)	−40%
Alcohol use disorders	631 (553–730)	634 (551–736)	0.5%	191 (114–175)	207 (152–227)	8%
Neoplasms	494 (436–548)	369 (295–446)	−25%	428 (368–485)	263 (203−330)	−39%
Cirrhosis and other chronic liver diseases	326 (278–369)	213 (178–249)	−34%	116 (95–137)	74 (59–89)	−36%
Cardiovascular diseases	525 (115–867)	139 (−66–355)	−74%	49 (−150–244)	−15 (−115–97)	−130%
Self‐harm	338 (201–465)	186 (78–283)	−45%	85 (30−130)	46 (7–81)	−45%
Unintentional injuries	162 (53–288)	125 (40–239)	−23%	88 (27–177)	73 (27–143)	−17%
*Sweden*	2000	2017	2000–2017	2000	2017	2000–2017
All alcohol‐attributed causes	1438 (1042–1895)	1161 (807–1587)	−19.3%	605 (382–838)	487 (275–756)	−19.6%
Alcohol use disorders	463 (388–559)	331 (260–416)	−29%	118 (92–153)	105 (76–150)	−11%
Neoplasms	235 (201–270)	204 (168–243)	−13%	270 (231–308)	205 (163–252)	−24%
Cirrhosis and other chronic liver diseases	119 (112–128)	101 (90–112)	−15%	36 (32–41)	43 (37–49)	17%
Cardiovascular diseases	5 (−297–309)	31 (−156–238)	523%	−19 (−180–146)	−14 (−122–98)	24%
Self‐harm	237 (90–361)	192 (62–311)	−19%	85 (27–137)	67 (15–115)	−20%
Unintentional injuries	127 (46–238)	128 (48–244)	1%	77 (29–149)	80 (29–154)	3%
*Norway*	2000	2017	2000–2017	2000	2017	2000–2017
All alcohol‐attributed causes	1102 (729–1518)	976 (868–1301)	−11.4%	239 (66–422)	270 (130–431)	13.4%
Alcohol use disorders	466 (396–554)	354 (285–419)	−26%	129 (101–164)	125 (98–159)	−3.2%
Neoplasms	208 (171–245)	174 (136–215)	−17%	197 (161–235)	145 (109–181)	−26%
Cirrhosis and other chronic liver diseases	104 (96–113)	81 (71–91)	−22%	39 (35–43)	31 (27–35)	−21%
Cardiovascular diseases	−272 (−562–16)	−62 (−200–82)	77%	−216 (−349–90)	−96 (−163‐ ‐28)	56%
Self‐harm	218 (53–362)	153 (41–249)	−30%	49 (2–94)	42 (2–79)	−14%
Unintentional injuries	152 (58–282)	137 (52–249)	−10%	77 (25–147)	75 (23–144)	1.7%

DALY, disability‐adjusted life‐years.

Norwegian males and females had the lowest alcohol‐attributed disease burden. In males, DALYs decreased from 1102 (95% UIs 729, 1518) in 2000 to 976 (95% UIs 868, 1301) in 2017 with a peak in 2004, corresponding to a decline by 11%. In Norwegian females, DALYs increased by 13%, from 239 (95% UIs 66, 422) in 2000 to 270 (95% UIs 130, 431) in 2017. This increase was mainly explained by a decrease in the protective effect of alcohol in cardiovascular diseases (Table S1).

The alcohol‐attributed diseases burden in Danish males decreased by 34%, from 2971 DALYs (95% UIs 2464, 3468) in 2000 to 1956 (95% UIs 1456, 2505) in 2017. Although their DALYs declined by 40%, from 1030 (95% UIs 748, 1334) in 2000 to 620 (95% UIs 358, 946) in 2017, Danish females have the highest alcohol‐attributed disease burden among females in the Nordic countries. In both males and females, the levels were highest in 2002–2004.

In Swedish males, the alcohol‐attributed disease burden decreased from 1438 DALYs (95% UIs 1042, 1895) in 2000 to 1161 (95% UIs 807, 1587) in 2017, and in Swedish females from 605 (95% UIs 382, 838) in 2000 to 487 (95% UIs 275, 756) in 2017. This corresponds to 19% and 20% declines in males and females, respectively. Levels in both males and females were highest in 2004.

Despite country differences in levels of alcohol‐attributed disease burden, males and females in all countries followed a similar pattern, in which alcohol use disorder, neoplasms, self‐harm, cirrhosis and unintentional injuries were the leading causes of DALYs (Figure 1 and Table 1). Together, these causes accounted for the majority of the alcohol‐attributed disease burden in 2017. In females, alcohol‐attributed disease burden caused by neoplasms was mainly driven by breast cancer (Table 1). Cardiovascular diseases were also important contributors to disease burden in Finnish and Danish males.

### *Gender differences in all‐cause alcohol‐attributed diseases burden and changes over time*

**Figure 2:**
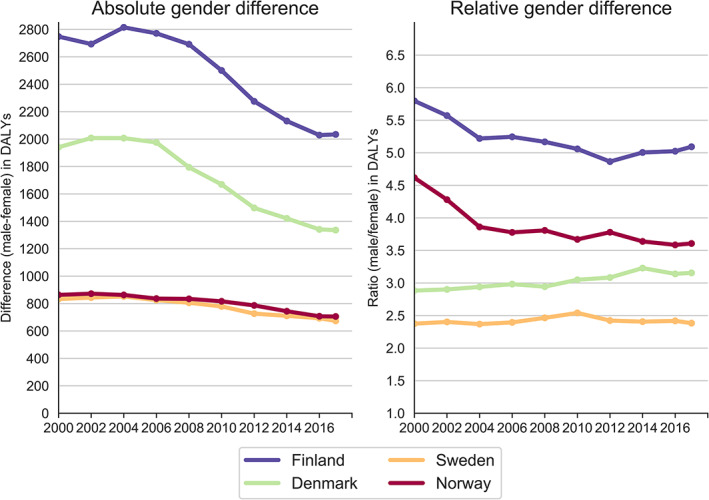
Absolute and relative gender differences in overall alcohol‐attributed disease burden by age‐standardised disability‐adjusted life‐years rates per 100 000 in the Nordic countries between 2000 and 2017.

Finland had the largest absolute gender differences in all‐cause DALYs over the study period, and Norway had the smallest, closely followed by Sweden (Figure 2 and Table S3). The gender differences in Denmark were closer to those in Finland. When comparing the years 2000 and 2017, the absolute differences in all‐cause DALYs declined by 31% in Denmark, 26% in Finland, 19% in Sweden and 18% in Norway.

In Finland, the decline in absolute gender differences in all‐cause alcohol‐attributed DALYs were due to a more pronounced decline among males than females. In Norway, the gender convergence was due to an increase among females and a decrease among males. In Denmark, on the other hand, the decline was substantial for both males and females, but slightly more marked in females. This means that gender differences, in relative terms, increased slightly in Danes between 2000 and 2017 (Figure 2 and Table S3). In Sweden, the all‐cause decline was similar in males and females over time, resulting in a rather stable gender gap over time.

### *Gender differences in cause‐specific alcohol‐attributed disease burden and changes over time*

**Figure 3:**
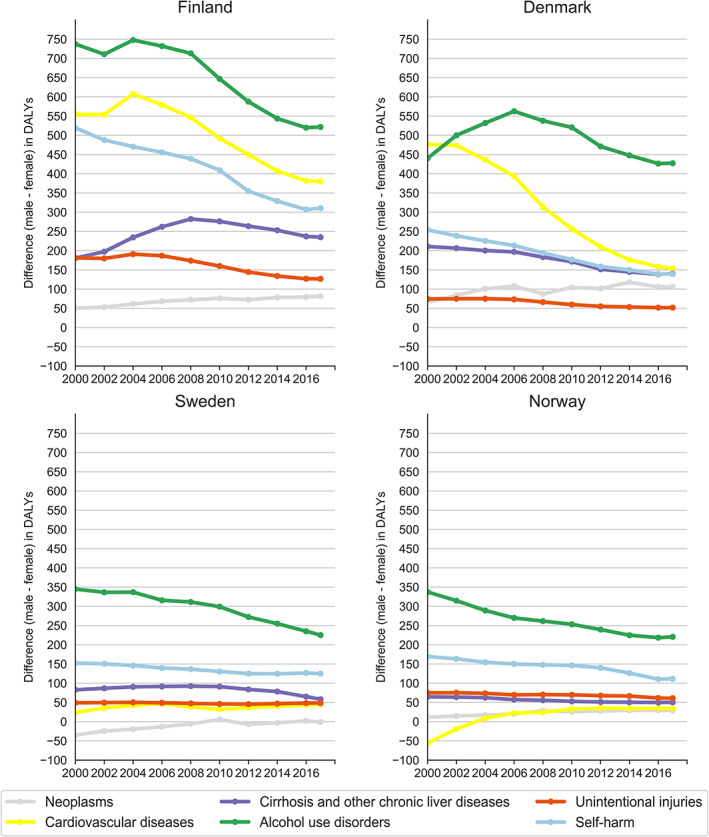
Absolute gender differences in cause‐specific alcohol‐attributed disease burden by age‐standardised disability‐adjusted life‐years rates per 100 000 in the Nordic countries between 2000 and 2017.

In Finland, the gender gap in alcohol use disorders, cardiovascular diseases, self‐harm and unintentional injuries decreased over time (Figure 3 and Appendix Table 3). While the burden from these causes declined in both genders, the decline was larger in males than in females. In contrast, gender differences in cirrhosis and neoplasms increased between 2000 and 2017. For neoplasms, this was due to a larger decline in females (25%) than in males (7%), while for cirrhosis this was due to a larger increase in males (25%) than in females (11%).

In Norway, the gender gap in alcohol use disorder and self‐harm decreased over time. For alcohol use disorder, this was explained solely by a decrease in males, and for self‐harm by a larger decrease in males than in females. Like in Finland, gender differences in neoplasms increased over time due to a larger decline in females (26%) than in males (16%). The burden from unintentional injuries was stable in both males and females, and cirrhosis declined equally in both genders. Consequently, the gender gap did not change for these causes. The estimated protective effect from alcohol on cardiovascular diseases has decreased more in males than in females.

In Denmark, the burden from alcohol use disorder did not decline in males between 2000 and 2017, and increased slightly in females. Consequently, the gender gap for alcohol use disorder was relatively stable. Self‐harm and cirrhosis declined to a similar degree in males and females. Gender differences in neoplasms increased between 2000 and 2017 due to a larger decrease in females (39%) than in males (25%). The burden from unintentional injuries was the only cause that decreased slightly more in males than in females.

In Sweden, the gender gap in the burden from alcohol use disorder decreased due to a larger decline in males than females. There was a similar decline in males and females for self‐harm, while cirrhosis increased in females and decreased in males. As in the other Nordic countries, gender differences in neoplasms increased between 2000 and 2017, and this was due to a larger decrease in females (24%) compared to males (13%).

## Discussion

Among males in the Nordic countries, Finns had the highest alcohol‐attributed disease burden and Norwegians the lowest. Among females, the burden was highest in Denmark and lowest in Norway. Finland had the largest gender differences and Norway had the smallest, closely followed by Sweden. The gender differences in Denmark were closer to those in Finland. The all‐cause DALYs declined in males and females in all countries except in Norwegian females. Alcohol use disorder, neoplasms, self‐harm, cirrhosis and unintentional injuries were the leading causes collectively accounting for the majority of DALYs among both males and females in all countries. In Finland and Denmark, cardiovascular diseases also made significant contributions to the disease burden. In Finland and Norway, the gender gap in overall alcohol‐attributed DALYs narrowed over time. In Finland, this was driven by a larger decline in males than females, and in Norway by an increased burden in females. In Denmark, on the other hand, the gender gap increased slightly, and although there was a decline in both males and females, the decline was slightly more marked in females. In Sweden, the decline was similar in males and females and therefore the relative gender gap was rather stable over time.

Comparative studies focusing on gender differences and assessing all diseases causally linked to alcohol are lacking in the Nordic countries. Thus, findings from prior studies do not enable an overall comparison of gender differences in the health burden gap from alcohol over time. However, the country and gender differences that we found point in the same direction as previous studies showing higher levels of alcohol consumption [6, 27, 28] and alcohol‐related mortality [22, 28, 29] among both genders in Finland and Denmark compared with in Sweden and Norway. Moreover, a gender convergence has been observed with increased alcohol use in Norwegian females [13]. In our study, the gender convergence in Norway was mainly explained by a decrease in the negative disease burden (i.e. the protective effect) for cardiovascular diseases in females. This negative burden may seem surprising, but reflects cardio‐protective effects from low to moderate consumption [2].

To a certain extent, the gender differences and trends that we have observed may reflect broader gender inequalities in society. The Nordic countries have long been international leaders on gender equality, and gender gaps in labour market participation and employment are among the smallest in Organisation for Economic Co‐operation and Development countries, particularly for highly educated males and females [32]. While gender inequalities remain, with males still having higher positions, higher income and being less likely to take parental leave [32], the decline in all‐cause disease burden in both males and females in all countries, except for Norwegian females, suggests that other factors may be more important in this context.

From an alcohol policy perspective, pricing, taxation and restricted availability are effective strategies to reduce alcohol consumption and related harm [20], and these tools remain in place, especially in Norway and Sweden. In Finland, the alcohol policies were considerably liberalised after the country joined the European Union in the mid‐1990s and alcohol taxes were strongly cut in 2004. In Denmark, the high taxation on spirits was lowered in the beginning of 2000 and in 2004, and the European Union's travellers' allowance for alcohol import for personal use became unlimited in Denmark, Finland and Sweden, resulting in a number of changes affecting both price and availability [21], and increasing alcohol‐related deaths, especially in Finland [33]. While several studies have analysed the effect of specific alcohol policy changes in relation to consumption and health outcomes in the Nordic countries, these types of studies in general require more detailed time‐specific data than we have provided. However, several alcohol tax increases after 2007 may explain the positive development that we have observed. Moreover, similar trends with increases in disease burden during 2000 and 2004 in our study, for both males and females, highlight the importance of the policies mentioned above.

At the same time, the large gender gaps over time, especially in Finland and Denmark, show that other factors are involved. In Finland, for example, the gender gap in disease burden over time might relate to a tradition of heavy drinking among males [34]. In a recent study, Finnish males also reported, somewhat more than females did, almost any kind of motivation to drink, such as liking the feeling of alcohol and getting drunk, to relax from work stress, and that it improved the atmosphere at events [35]. In Sweden, another study showed that males were more likely than females to get drunk in order to have fun at events and to fit into a group, but that the proportions of females and males reporting that they drank for pleasure were equal [36]. Although no general conclusions can be drawn, these kinds of socio‐cultural differences may to some extent help explain the larger gender gaps in disease burden of alcohol in Finland compared with Sweden. However, the declining trends in DALYs, also in males, suggest that drinking norms and attitudes towards alcohol may have changed, at least in some groups. For instance, it has been found that heavy drinking, the traditional male drinking style in the Nordic countries, is not as popular in males with high education [37, 38].

Another important factor is thus how socioeconomic inequalities relate to alcohol‐related harm in males and females [39]. The Nordic countries are comparably egalitarian welfare states with generous welfare policies. However, there are inequalities in life expectancies by income levels [28, 40]. These inequalities have in part been explained by socioeconomic differences in alcohol‐related mortality in both males and females [28]. The rates of alcohol‐related mortality are higher in lower socioeconomic groups, even if those in lower socioeconomic groups report equivalent or less alcohol consumption than those in higher groups. The reason for this ‘alcohol harm paradox’ presumably relates to other underlying risk factors, such as patterns of drinking, smoking habits, poorer quality of health care and less social support that may lead to elevated risks of alcohol in lower socioeconomic groups [41]. How contexts and policy influence alcohol use and related harm by gender in various socioeconomic groups is an important question but remains largely unknown [42]. A study from Finland showed that changes in alcohol policy, such as reduced prices, may have the largest impact on males in lower socioeconomic groups, when it comes to severe alcohol‐related harm [43]. To date, however, estimates of disease burden by socioeconomic levels in the GBD are not yet available within countries and therefore could not be investigated further in our study.

Information about the cause‐specific alcohol‐attributed disease burden and the narrowing gender gap in most alcohol‐attributed causes may have important clinical implications, particularly given the apparent convergence for some causes observed in our study. For example, while males had a slightly higher burden from neoplasms than females in Finland, Denmark and Norway, the burden from neoplasms had decreased more in females than in males, in all countries. The decline in females is primarily explained by a decrease in premature mortality (YLL) from breast cancer (data not shown).

It is noteworthy that females in Denmark and Norway seem to have lagged behind with regards to the declining burden from alcohol use disorder, while health gains were being made by both genders in Finland, Sweden and among males in Norway. In Danish males, the burden had not changed, and the slight increase in Danish females can mainly be explained by increased premature deaths (YLLs) in alcohol use disorder (data not shown). Hence, our results provide targets for nations like Denmark to improve survival rates in females with alcohol use disorder. There is also the possibility of differences in time lags with regards to the health effects of alcohol consumption. However, these lags will not differ between countries and gender. Thus, continued improvements in use of medical care and preventive measures will be necessary in both males and females to achieve further reductions related to the alcohol‐attributed disease burden in the Nordic countries.

Limitations of the GBD methodology in relation to measurements of the alcohol‐attributed disease burden have been described in detail previously [2]. For example, per‐capita consumption is often underestimated and the quantification of consumption from sales data may not fully capture illicit production or unrecorded consumption. Drinking patterns were not taken into account in the calculation of disease burden, despite its importance for some diseases. Moreover, the risk of alcohol use has only been estimated for outcomes with evidence meeting the criteria of the GBD. There may be additional outcomes, such as depression for example [44], for which alcohol might be a risk factor. Taken together, this may limit the estimations of the alcohol‐attributed disease burden. However, this should not differ systematically between countries.

In addition, the quality of disease and mortality data in each country will also affect the estimated burden attributed to alcohol, since estimates rely on these data as well. For example, fatal outcomes are based on cause of death registers, which are generally of high quality in the Nordic countries. While coding‐practices for cardiovascular diseases and cancers are rather stable across the Nordic countries, they tend to differ for alcohol‐attributed deaths [45]. Little is known about the extent to which differences in coding affect the estimates between these countries. However, the GBD study uses a standardised approach to assess causes of death for all countries, for example, unspecified International Classification of Diseases and Related Health problems codes that cannot be underlying causes of deaths are redistributed into valid death codes using algorithms, to improve internal comparability.

With regard to non‐fatal outcomes, these are mainly based on scientific studies and registers reflecting country prevalence of diseases. The number of scientific studies and underlying data in these registers are more sparse and can be incomplete and untimely, and coding practices may, as for the cause of death registers, differ between these countries [45]. Hence, estimates should be interpreted with caution. It should also be mentioned that the GBD methodology differs from that in many national studies in this area and that GBD uses many different sources to reflect country prevalence of disease and exposure, which is why results may differ from those found in national studies. DALYs are also the results of very sophisticated modelling that may, to some extent, be distanced from the underlying data.

It should be noted that the declining trends for many causes could to some extent be influenced by other underlying factors, such as improved treatment or reduced smoking for example. On the other hand, the population attributable burden is an expression of the percentage reduction of health outcomes in the population that occur if exposure to a specific risk factor, such as alcohol, should decrease. The GBD study calculates the population attributable burden in males and females for every risk factor and cause separately within a comprehensive and comparable framework. This means that the declining burden for a given cause attributed to other risk factors than alcohol should therefore not impact the results of this study.

Despite these limitations, the GBD study offers the most comprehensive and comparative framework that refines its estimates as new data and methods become available [46]. To conclude, our study showed that there were considerable differences in alcohol‐attributed DALYs between the Nordic countries and by gender. The all‐cause DALYs declined in males and females in all countries except in Norwegian females, and gender gaps in harm narrowed to a different extent and was driven by different conditions. In Finland and Norway, the gender gap narrowed between 2000 and 2017. In Finland, this was driven by larger relative decline in males than in females, and in Norway due to increased burden in females. In Denmark, the gap slightly increased and in Sweden it was rather stable. In Denmark, this was driven by a slightly larger decline in females than in males and in Sweden, by similar declines in males and females. These findings are informative about how gender inequality, policy and sociocultural differences may affect levels of harm by gender over time. However, to gain a deeper understanding of gender differences in the all‐cause and cause‐specific burden of alcohol across these countries, as well as the impact of policy measures, the influence of socioeconomic inequalities within countries requires further investigation.

## Conflict of interest

The authors have no conflicts of interest.

## Supporting information


**Table S1.** Alcohol‐attributed disease burden in age‐standardised DALY rates per 100 000 in four Nordic countries 2000 and 2017, and percentage change 2000‐2017 by all‐cause and cause‐specific disease burden.
**Table S2**. Detailed diagnoses of causes attributed to alcohol Global Burden of Disease study 2017.
**Table S3**. Alcohol‐attributed disease burden in age‐standardised DALY rates per 100 000 in four Nordic countries 2000 and 2017, with absolute and relative gender differences by all‐cause and cause‐specific disease burden.
