# Supplementary material for: Alcohol‐attributed disease burden in four Nordic countries between 2000 and 2017: Are the gender gaps narrowing? A comparison using the Global Burden of Disease, Injury and Risk Factor 2017 study
Source: Drug Alcohol Rev. 2020 Nov 18;40(3):431–42. doi: 10.1111/dar.13217 (PMC7983874; doi:10.1111/dar.13217)
Supplement: Supplementary file 1 — Table S1. Alcohol‐attributed disease burden in age‐standardised DALY rates per 100 000 in four Nordic countries 2000 and 2017, and percentage change 2000‐2017 by all‐cause and cause‐specific disease burden. Table S2. Detailed diagnoses of causes attributed to alcohol Global Burden of Disease study 2017. Table S3. Alcohol‐attributed disease burden in age‐standardised DALY rates per 100 000 in four Nordic countries 2000 and 2017, with absolute and relative gender differences by all‐cause and cause‐specific disease burden. [file DAR-40-431-s001.docx]

Table S1. Alcohol-attributed disease burden in age-standardised DALY rates per 100 000 in four Nordic countries 2000 and 2017, and percentage change 2000-2017 by all cause and cause-specific disease burden

|  | **Males** | | | **Females** | | |
| --- | --- | --- | --- | --- | --- | --- |
|  | **Age-standardised DALYs per 100.000 with 95% uncertainty intervals** | | **Percent change in age-standardised DALYs per 100.000** | **Age-standardised DALYs per 100.000 with 95% uncertainty intervals** | | **Percent change in age-standardised DALYs per 100.000** |
| **Finland** | **2000** | **2017** | **2000-2017** | **2000** | **2017** | **2000-2017** |
| **All alcohol-attributed causes** | **3346 (2709-4067)** | **2531 (1983-3192)** | **-24%** | **578 (346-830)** | **497 (309-716)** | **-13%** |
| Alcohol use disorders | 1005 (873-1159) | 739 (616-844) | -27% | 268 (202-327) | 217 (169-270) | -19% |
| *Neoplasms* | 244 (217-272) | 227 (191-268) | -7% | 194 (164-222) | 146 (114-180) | -25% |
| Oesophageal cancer | 44 (36-51) | 41 (33-50) | -5% | 9 (5-12) | 7 (4-9) | -26% |
| Liver cancer | 44 (37-52) | 50 (40-61) | 12% | 13 (10-17) | 12 (9-15) | -9% |
| Larynx cancer | 13 (9-17) | 9 (5-12) | -33% | 0.94 (0.3-1.6) | 0.6 (0.15-1.1) | -33% |
| Breast cancer | 2 (1-8) | 1 (1-1) | -24% | 128 (104-151) | 91 (67-114) | -29% |
| Colon and rectum cancer | 87 (66-108) | 75 (52-101) | -13% | 29 (17-41) | 23 (13-35) | -20% |
| Lip and oral cavity cancer | 31 (27-34) | 27 (22-32) | -12% | 8 (6-11) | 8 (5-11) | -7% |
| Pharynx and nasopharynx cancer | 8 (7-10) | 6 (5-7) | -29% | 7 (1-2) | 1 81-1) | -38% |
| Cirrhosis and other chronic liver diseases | 251 (209-287) | 313 (248-373) | 25% | 70 (56-85) | 78 (61-95) | 11% |
| *Cardiovascular diseases* | 380 (-40-775) | 213 (33-616) | -18% | -175 (-325- -19) | -67 (-148-23) | 61% |
| Ischaemic heart disease | -78 (-449-278) | -54 (-279-182) | 32% | -183 (-317- -47) | -90 (-155- -27) | 51% |
| Ishcaemic stroke | 106 (36-175) | 61 (13-113) | -42% | -55 (-123-6) | -34 (-75-6) | 39% |
| Haemorrhagic stroke | 108 (76-139) | 69 (41-96) | -36% | 22 (-9-49) | 13 (-6-30) | -41% |
| Hypertensive heart disease | 28 (21-44) | 49 (22-65) | 74% | 13 (6-20) | 16 (6-26) | 27% |
| Atrial fibrillation and flutter | 31 (21-43) | 32 (20-48) | 20% | 9 (5-14) | 8 (4-13) | -6% |
| Diabetes mellitus | 68 (19-124) | 72 (12-139) | 7% | -88 (-164- -33) | -96 (-181- -29) | -9% |
| Self-harm | 646 (380-874) | 377 (214-534) | -42% | 102 (16-177) | 66 (11-118) | -35% |
| Interpersonal violence | 69 (45-93) | 39 (24-56) | -42% | 21 (9-32) | 13 (6-21) | -37% |
| Transport injuries | 167 (97-218) | 104 (62-134) | -38% | 37 (14-63) | 26 (9-45) | -28% |
| Unintenttional injuries | 280 (94-494) | 206 (70-374) | -26% | 98 (35-188) | 97 (27-156) | -19% |
| Epilepsy | 68 (41-113) | 56 (31-99) | -18% | 27 (11-56) | 25 (9-51) | -7% |
| Pancreatitis | 75 (57-91) | 66 (43-87) | -12% | 6 (0.4-12) | 6 (-1-12) | -11.5% |
| Lower respiratory infections | 76 (12-132) | 15 (2-28) | -81% | 6 (-18-28) | 1.2 (-3-5) | -79% |
| Tuberculosis | 19 (15-22) | 6 (4-7) | -69% | 6 (4-9) | 3 (1-4) | -61% |
| **Denmark** | **2000** | **2017** | **2000-2017** | **2000** | **2017** | **2000-2017** |
| **All alcohol-attributed causes** | **2971 (2464-3468)** | **1956 (1456-2505)** | **-34%** | **1030 (748-1334)** | **620 (358-946)** | **-40%** |
| Alcohol use disorders | 631 (553-730) | 634 (551-736) | 0.5% | 191 (114-175) | 207 (152-227) | 8% |
| *Neoplasms* | 494 (436-548) | 369 (295-446) | -25% | 428 (368-485) | 263 (203-330) | -39% |
| Oesophageal cancer | 99 (84-112) | 75 (58-92) | -23% | 25 (19-30) | 16 (10-22) | -35% |
| Liver cancer | 46 (39-53) | 55 (44-67) | 20% | 14 (11-19) | 14 (10-19) | -2% |
| Larynx cancer | 33 (24-40) | 18 (10-24) | -47% | 5 (3-7) | 3 (1-4) | -53% |
| Breast cancer | 3 (2-4) | 2 (1-2) | -41% | 250 (205-290) | 145 (110-185) | -42% |
| Colon and rectum cancer | 190 (145-230) | 117 (75-165) | -38% | 97 (65-127) | 58 (33-87) | -40% |
| Lip and oral cavity cancer | 58 (52-63) | 41 (33-50) | -29% | 17 (14-20) | 13 (9-17) | -27% |
| Pharynx and nasopharynx cancer | 19 (16-21) | 13 (10-16) | -30% | 4 (3-4) | 2 (1-3) | -41% |
| Cirrhosis and other chronic liver diseases | 326 (278-369) | 213 (178-249) | -34% | 116 (95-137) | 74 (59-89) | -36% |
| *Cardiovascular diseases* | 525 (115-867) | 139 (-66-355) | -74% | 49 (-150-244) | -15 (-115-97) | -130% |
| Ischaemic heart disease | 86 (-259-380) | -66 (-216- -95) | -182% | -106 (-274- -66) | -62 (-129- -2) | 41% |
| Ishcaemic stroke | 123 (69-173) | 50 (1-93) | -60% | 21 (-35- -72) | -12 (-59- -34) | -156% |
| Haemorrhagic stroke | 197 (144-244) | 81 (42-120) | -59% | 96 (49-140) | 34 (-0.2-64) | -65% |
| Hypertensive heart disease | 21 (15-26) | 12 (8-16) | -43% | 12 (7-17) | 7 (4-11) | -45% |
| Atrial fibrillation and flutter | 49 (33-67) | 36 (22-53) | -26% | 21 (14-28) | 15 (8-22) | -30% |
| Diabetes mellitus | 108 (35-188) | 75 (-26-170) | -31% | -51 (-163-50) | -112 (-232-11) | -120% |
| Self-harm | 338 (201-465) | 186 (78-283) | -45% | 85 (30-130) | 46 (7-81) | -45% |
| Interpersonal violence | 33 (20-46) | 20 (10-30) | -39% | 17 (8-26) | 10 (4-15) | -44% |
| Transport injuries | 190 (113-242) | 83 (43-110) | -56% | 45 (19-73) | 30 (11-49) | -35% |
| Unintenttional injuries | 162 (53-288) | 125 (40-239) | -23% | 88 (27-177) | 73 (27-143) | -17% |
| Epilepsy | 55 (31-94) | 46 (21-83) | -17% | 33 (15-62) | 27 (10-57) | -19% |
| Pancreatitis | 49 (37-60) | 18 (15-41) | 43% | 12 (6-17) | 8 (1-14) | -33% |
| Lower respiratory infections | 48 (12-79) | 33 (-6-74) | -31% | 13 (-7-30) | 8 (-13-26) | -40% |
| Tuberculosis | 11 (9-13) | 5 (4-7) | -53% | 5 (4-6) | 2 (1-3) | -58% |
| **Sweden** | **2000** | **2017** | **2000-2017** | **2000** | **2017** | **2000-2017** |
| **All alcohol-attributed causes** | **1438 (1042-1895)** | **1161 (807-1587)** | **-19.3 %** | **605 (382-838)** | **487 (275-756)** | **-19.6 %** |
| Alcohol use disorders | 463 (388-559) | 331 (260-416) | -29% | 118 (92-153) | 105 (76-150) | -11% |
| *Neoplasms* | 235 (201-270) | 204 (168-243) | -13% | 270 (231-308) | 205 (163-252) | -24% |
| Oesophageal cancer | 42 (33-50) | 38 (27-47) | -11% | 14 (10-17) | 11 (7-14) | -24% |
| Liver cancer | 53 (49-56) | 53 (47-60) | 1% | 16 (14-18) | 14 (12-16) | -16% |
| Larynx cancer | 8 (4-10) | 5 (2-7) | -34% | 2 81-2) | 1 (0-2) | -38% |
| Breast cancer | 1 81-2) | 1 81-1) | -25% | 162 (135-190) | 119 (93-147) | -26% |
| Colon and rectum cancer | 90 (62-116) | 69 (45-96) | -23% | 60 (41-80) | 46 (28-67) | -24% |
| Lip and oral cavity cancer | 21 (16-24) | 18 (13-22) | -13% | 10 (8-12) | 9 (6-11) | -10% |
| Pharynx and nasopharynx cancer | 7 (5-8) | 5 (4-6) | -23% | 2 (1-2) | 1 (1-1) | -37% |
| Cirrhosis and other chronic liver diseases | 119 (112-128) | 101 (90-112) | -15% | 36 (32-41) | 43 (37-49) | 17% |
| *Cardiovascular diseases* | 5 (-297-309) | 31 (-156-238) | 523% | -19 (-180-146) | -14 (-122-98) | 24% |
| Ischaemic heart disease | -228 (-503-35) | -136 (-290-21) | 41% | -111 (-247-24) | -69 (145-6) | 38% |
| Ishcaemic stroke | 54 (0-106) | 28 (-14-66) | -48% | 4 (-52-54) | -6 (-47-32) | -266% |
| Haemorrhagic stroke | 89 (51-125) | 49 (23-74) | -45% | 53 (20-82) | 26 (5-46) | -50% |
| Hypertensive heart disease | 12 (8-16) | 17 (8-24) | 51% | 10 (6-14) | 14 (5-21) | 36% |
| Atrial fibrillation and flutter | 32 (21-46) | 32 (17-49) | -2% | 19 (12-26) | 17 (10-26) | -11% |
| Diabetes mellitus | 31 (-19-78) | 24 (-38-80) | -24% | -49 (-114-12) | -65 (-145-9) | -32% |
| Self-harm | 237 (90-361) | 192 (62-311) | -19% | 85 (27-137) | 67 (15-115) | -20% |
| Interpersonal violence | 28 (15-41) | 26 (13-49) | -6% | 13 (6-19) | 10 (4-16) | -22% |
| Transport injuries | 112 (55-156) | 66 (29-94) | -41% | 31 (13-50) | 23 (9-39) | -25% |
| Unintentional injuries | 127 (46-238) | 128 (48-244) | 1% | 77 (29-149) | 80 (29-154) | 3% |
| Epilepsy | 38 (22-64) | 28 (14-49) | -26% | 24 (11-46) | 21 (9-41) | -10% |
| Pancreatitis | 14 (9-20) | 12 (5-19) | -14% | 5 (2-7) | 4 (1-8) | -6% |
| Lower respiratory infections | 23 (-5-48) | 15 (-5-35) | -34% | 9 (-7-23) | 5 (-5-15) | -42% |
| Tuberculosis | 8 (6-9) | 3 (2-4) | -55% | 6 (4-7) | 2 (2-3) | -57% |
| **Norway** | **2000** | **2017** | **2000-2017** | **2000** | **2017** | **2000-2017** |
| **All alcohol-attributed causes** | **1102 (729-1518)** | **976 (868-1301)** | **-11.4%** | **239 (66-422)** | **270 (130-431)** | **13.4%** |
| Alcohol use disorders | 466 (396-554) | 354 (285-419) | -26% | 129 (101-164) | 125 (98-159) | -3.2% |
| *Neoplasms* | 208 (171-245) | 174 (136-215) | -17% | 197 (161-235) | 145 (109-181) | -26% |
| Oesophageal cancer | 34 (24-43) | 30 (21-39) | -11% | 7 (4-10) | 6 (3-8) | -19% |
| Liver cancer | 25 823-28) | 29 (25-32) | 12% | 8 (7-9) | 8 (7-10) | 9% |
| Larynx cancer | 9 (4-13) | 6 (2-8) | -37% | 1 (0-2) | 0.7 (0-1) | -31% |
| Breast cancer | 1.5 (1-2) | 1 (1-1) | -28% | 121 (95-147) | 82 (61-105) | -32% |
| Colon and rectum cancer | 88 (59-115) | 72 (45-101) | -18% | 46 (24-66) | 37 (19-54) | -19% |
| Lip and oral cavity cancer | 27 (20-32) | 18 (13-23) | -32% | 8 (5-10) | 6 (4-8) | -19% |
| Pharynx and nasopharynx cancer | 9 (7-11) | 6 (5-8) | -31% | 2 (1-2) | 1 (1-2) | -33% |
| Cirrhosis and other chronic liver diseases | 104 (96-113) | 81 (71-91) | -22% | 39 (35-43) | 31 (27-35) | -21% |
| *Cardiovascular diseases* | -272 (-562-16) | -62 (-200-82) | 77% | -216 (-349-90) | -96 (-163- -28) | 56% |
| Ischaemic heart disease | -399 (-660- -119) | -154 (-268- -40) | 61% | -189 (-310- -37) | -83 (-135- -31) | 56% |
| Ishcaemic stroke | 10 (-45-61) | 11 (-28-48) | 19% | -61 (-109- -16) | -38 (-70- -6) | 37% |
| Haemorrhagic stroke | 68 (22-11) | 36 (11-59) | -47% | 16 (-21-51) | 10 (-11-30) | -38% |
| Hypertensive heart disease | 7 (5-11) | 6 (3-9) | -22% | 4 (1-7) | 4 (1-7) | -12% |
| Atrial fibrillation and flutter | 21 (13-30) | 24 (14-36) | 11% | 8 (4-12) | 9 (5-14) | 10% |
| Diabetes mellitus | -7 (-55-40) | -0.1 (-53-52) | 98% | -107 (-171- -55) | -110 (-176- -55) | -2.8% |
| Self-harm | 218 (53-362) | 153 (41-249) | -30% | 49 (2-94) | 42 (2-79) | -14% |
| Interpersonal violence | 22 (10-33) | 16 (7-24) | -28% | 10 (2-17) | 8 (2-13) | -24% |
| Transport injuries | 135 (59-195) | 69 (33-98) | -49% | 28 (11-47) | 20 (8-34) | -27% |
| Unintentional injuries | 152 (58-282) | 137 (52-249) | -10% | 77 (25-147) | 75 (23-144) | 1.7% |
| Epilepsy | 43 (24-70) | 39 (21-64) | -9% | 24 (9-45) | 24 (8-44) | -3% |
| Pancreatitis | 11 (4-18) | 9 (3-16) | -12% | 3 (-1.6-7) | 3 (-1-7) | -7% |
| Lower respiratory infections | 13 (-23-47) | 11 (-13-32) | -20% | 4 (-13-20) | 2 (-8-13) | -31% |
| Tuberculosis | 8 (6-10) | 4 (2-5) | -56% | 4 (2-5) | 2 81-3) | -42% |

DALY, disability-adjusted life-years.

Table S2: Detailed diagnoses of causes attributed to alcohol Global Burden of Disease study 2017

| *A: Injuries* |  |
| --- | --- |
| *A.1 Transport injuries* |  |
| *A.1.1. Road injuries* | Pedestrian road injuries; cyclist road injuries; motorcyclist road injuries; motor vehicle road injuries; other road injuries. |
| *A.1.2. Other transport injuries* |  |
| *A.2 Unintentional injuries* | Falls; drowning; fire; heat and hot substances; poisonings by carbon monoxide; poisoning by other means; unintentional firearm injuries; venomous animal contact; non-venomous animal contact; environmental heat and cold exposure; exposures to forces of nature; other unintentional injuries. |
| *B: Self-harm and interpersonal violence* |  |
| *B.1. Self-harm* | Self-harm by firearm; self-harm by other specified means. |
| *B.2. Interpersonal violence* | Assault by firearm; assault by sharp object; sexual violence; assault by other means. |
| C. Respiratory infections and tuberculosis |  |
| *C.1* | Drug-susceptible tuberculosis. multidrug-resistant tuberculosis without extensive drug resistance; extensively drug-resistant tuberculosis. |

Table S3. Alcohol-attributed disease burden in age-standardised DALY rates per 100 000 in four Nordic countries 2000 and 2017, with absolute and relative gender differences by all cause and cause-specific disease burden

| **Finland** | | | | |
| --- | --- | --- | --- | --- |
| **All alcohol-attributed causes** | Males | Females | Absolute difference* | Relative difference** |
| 2000 | 3321 | 573 | 2748 | 5.80 |
| 2002 | 3283 | 589 | 2694 | 5.57 |
| 2004 | 3482 | 667 | 2815 | 5.22 |
| 2006 | 3425 | 653 | 2772 | 5.25 |
| 2008 | 3338 | 646 | 2692 | 5.17 |
| 2010 | 3117 | 616 | 2501 | 5.06 |
| 2012 | 2864 | 589 | 2275 | 4.86 |
| 2014 | 2664 | 532 | 2132 | 5.01 |
| 2016 | 2533 | 504 | 2029 | 5.03 |
| 2017 | 2531 | 497 | 2034 | 5.09 |
| *Percent change 2000 to 2017* | **-24%** | **-13%** | **-26%** | **0.12** |
| **Alcohol use disorder** | Males | Females | Absolute difference* | Relative difference** |
| 2000 | 1005 | 268 | 737 | 3.75 |
| 2002 | 978 | 267 | 711 | 3.66 |
| 2004 | 1022 | 275 | 748 | 3.72 |
| 2006 | 999 | 267 | 732 | 3.74 |
| 2008 | 973 | 260 | 713 | 3.74 |
| 2010 | 895 | 248 | 647 | 3.61 |
| 2012 | 825 | 237 | 588 | 3.48 |
| 2014 | 766 | 222 | 544 | 3.45 |
| 2016 | 738 | 218 | 520 | 3.39 |
| 2017 | 739 | 217 | 522 | 3.41 |
| *Percent change 2000 to 2017* | -26% | -19% | -29% | 0.09 |

| **Finland (cont)** |  |  |  |  |
| --- | --- | --- | --- | --- |
| **Neoplasms** | Males | Females | Absolute difference* | Relative difference† |
| 2000 | 244 | 194 | 51 | 1.26 |
| 2002 | 240 | 187 | 53 | 1.28 |
| 2004 | 254 | 192 | 61 | 1.32 |
| 2006 | 251 | 183 | 68 | 1.37 |
| 2008 | 252 | 179 | 72 | 1.41 |
| 2010 | 250 | 174 | 76 | 1.44 |
| 2012 | 240 | 167 | 73 | 1.44 |
| 2014 | 232 | 154 | 78 | 1.51 |
| 2016 | 226 | 146 | 80 | 1.55 |
| 2017 | 227 | 146 | 81 | 1.55 |
| *Percent change 2000 to 2017* | **-7%** | **-25%** | **59%** | **-0.23** |
| **Self-harm** | Males | Females | Absolute difference* | Relative difference† |
| 2000 | 646 | 102 | 544 | 6.33 |
| 2002 | 583 | 95 | 488 | 6.14 |
| 2004 | 573 | 102 | 471 | 5.62 |
| 2006 | 547 | 91 | 456 | 6.01 |
| 2008 | 532 | 92 | 439 | 5.78 |
| 2010 | 497 | 87 | 409 | 5.71 |
| 2012 | 437 | 82 | 355 | 5.33 |
| 2014 | 398 | 69 | 329 | 5.77 |
| 2016 | 372 | 65 | 307 | 5.72 |
| 2017 | 377 | 66 | 310 | 5.71 |
| *Percent change 2000 to 2017* | **-41%** | **-35%** | **-43%** | **0.1** |

| **Finland (cont)** |  |  |  |  |
| --- | --- | --- | --- | --- |
| **Cirrhosis** | Males | Females | Absolute difference* | Relative difference† |
| 2000 | 251 | 70 | 181 | 3.59 |
| 2002 | 273 | 76 | 197 | 3.59 |
| 2004 | 322 | 87 | 235 | 3.70 |
| 2006 | 354 | 92 | 262 | 3.85 |
| 2008 | 378 | 95 | 282 | 3.98 |
| 2010 | 370 | 93 | 276 | 3.98 |
| 2012 | 354 | 90 | 264 | 3.93 |
| 2014 | 336 | 83 | 253 | 4.05 |
| 2016 | 316 | 79 | 237 | 4.00 |
| 2017 | 313 | 78 | 235 | 4.01 |
| *Percent change 2000 to 2017* | **25%** | **11%** | **30%** | **-0.12** |
| **Unintentional injuries** | Males | Females | Absolute difference* | Relative difference† |
| 2000 | 280 | 98 | 181 | 2.86 |
| 2002 | 281 | 101 | 180 | 2.78 |
| 2004 | 296 | 105 | 191 | 2.82 |
| 2006 | 289 | 103 | 187 | 2.81 |
| 2008 | 273 | 99 | 174 | 2.76 |
| 2010 | 254 | 94 | 160 | 2.70 |
| 2012 | 234 | 90 | 144 | 2.60 |
| 2014 | 219 | 85 | 134 | 2.58 |
| 2016 | 208 | 81 | 127 | 2.57 |
| 2017 | 206 | 79 | 126 | 2.61 |
| *Percent change 2000 to 2017* | **-26%** | **-19%** | **-30%** | **0.09** |

| **Finland (cont)** |  |  |  |  |
| --- | --- | --- | --- | --- |
| **Cardiovascular diseases** | Males | Females† | Absolute difference* | Relative difference† |
| 2000 | 380 | -175 | 555 | -2.18 |
| 2002 | 403 | -150 | 554 | -2.68 |
| 2004 | 492 | -116 | 608 | -4.25 |
| 2006 | 484 | -96 | 579 | -5.05 |
| 2008 | 459 | -88 | 546 | -5.23 |
| 2010 | 413 | -79 | 492 | -5.22 |
| 2012 | 378 | -72 | 450 | -5.23 |
| 2014 | 342 | -65 | 407 | -5.26 |
| 2016 | 316 | -66 | 381 | -4.81 |
| 2017 | 313 | -67 | 380 | -4.64 |
| *Percent change 2000 to 2017* | **-18%** | **-61%** | **-32%** |  |

*Percent change in absolute gender differences between 2000 and 2017: 100*(male-female difference in DALY rates _2017_ - male-female difference in DALY rates _2000_ ) / (male-female differences in DALY rates _2000_). †Percent change in relative gender differences between 2000 and 2017: 1- (male/female ratio in DALY rates_2017_)/ (male/female ratio in DALY rates_2000_). ‡The protective effect from alcohol on cardiovascular diseases has decreased.

|  | **Denmark** | | | | |
| --- | --- | --- | --- | --- | --- |
|  | **All alcohol-attributed causes** | Males | Females | Absolute difference* | Relative difference** |
|  | 2000 | 2971 | 1030 | 1941 | 2.88 |
|  | 2002 | 3064 | 1056 | 2008 | 2.9 |
|  | 2004 | 3042 | 1035 | 2007 | 2.9 |
|  | 2006 | 2972 | 996 | 1976 | 2.98 |
|  | 2008 | 2717 | 923 | 1794 | 2.94 |
|  | 2010 | 2484 | 814 | 1670 | 3.49 |
|  | 2012 | 2216 | 719 | 1497 | 3.08 |
|  | 2014 | 2059 | 638 | 1421 | 3.22 |
|  | 2016 | 1968 | 627 | 1341 | 3.14 |
|  | 2017 | 1956 | 620 | 1336 | 3.15 |
|  | *Percent change 2000 to 2017* | **-34%** | **-39%** | **-31%** | **-0.09** |
|  | **Alcohol use disorder** | Males | Females | Absolute difference* | Relative difference** |
|  | 2000 | 631 | 191 | 440 | 3.32 |
|  | 2002 | 708 | 208 | 500 | 3.40 |
|  | 2004 | 751 | 219 | 532 | 3.43 |
|  | 2006 | 785 | 223 | 563 | 3.52 |
|  | 2008 | 763 | 225 | 538 | 3.39 |
|  | 2010 | 732 | 212 | 521 | 3.45 |
|  | 2012 | 675 | 204 | 471 | 3.31 |
|  | 2014 | 643 | 195 | 448 | 3.30 |
|  | 2016 | 632 | 205 | 426 | 3.08 |
|  | 2017 | 634 | 207 | 427 | 3.06 |
|  | *Percent change 2000 to 2017* | **0.4%** | **8%** | **-3%** | **0.08** |

|  | **Denmark (cont)** |  |  |  |  |
| --- | --- | --- | --- | --- | --- |
|  | **Neoplasms** | Males | Females | Absolute difference* | Relative difference** |
|  | 2000 | 494 | 428 | 66 | 1.15 |
|  | 2002 | 506 | 422 | 84 | 1.20 |
|  | 2004 | 496 | 395 | 101 | 1.26 |
|  | 2006 | 488 | 381 | 108 | 1.28 |
|  | 2008 | 454 | 366 | 88 | 1.24 |
|  | 2010 | 444 | 339 | 104 | 1.31 |
|  | 2012 | 408 | 306 | 102 | 1.33 |
|  | 2014 | 391 | 273 | 118 | 1.43 |
|  | 2016 | 372 | 266 | 106 | 1.40 |
|  | 2017 | 369 | 263 | 106 | 1.40 |
|  | *Percent change 2000 to 2017* | **-25%** | **-39%** | **61%** | **-0.21** |
|  | **Self-harm** | Males | Females | Absolute difference* | Relative difference** |
|  | 2000 | 338 | 85 | 254 | 3.98 |
|  | 2002 | 321 | 83 | 239 | 3.87 |
|  | 2004 | 301 | 76 | 226 | 3.96 |
|  | 2006 | 283 | 70 | 213 | 4.04 |
|  | 2008 | 260 | 66 | 194 | 3.94 |
|  | 2010 | 234 | 57 | 177 | 4.11 |
|  | 2012 | 209 | 50 | 159 | 4.18 |
|  | 2014 | 194 | 44 | 150 | 4.41 |
|  | 2016 | 185 | 46 | 139 | 4.02 |
|  | 2017 | 186 | 46 | 139 | 4.04 |
|  | *Percent change 2000 to 2017* | **-45%** | **-46%** | **-45%** | **-0.02** |

|  | **Denmark (cont)** |  |  |  |  |
| --- | --- | --- | --- | --- | --- |
|  | **Cirrhosis** | Males | Females | Absolute difference* | Relative difference** |
|  | 2000 | 326 | 116 | 211 | 2.81 |
|  | 2002 | 321 | 115 | 206 | 2.79 |
|  | 2004 | 313 | 113 | 200 | 2.77 |
|  | 2006 | 305 | 108 | 197 | 2.82 |
|  | 2008 | 286 | 102 | 183 | 2.80 |
|  | 2010 | 266 | 94 | 171 | 2.83 |
|  | 2012 | 235 | 84 | 152 | 2.80 |
|  | 2014 | 220 | 75 | 145 | 2.93 |
|  | 2016 | 212 | 74 | 139 | 2.86 |
|  | 2017 | 214 | 74 | 140 | 2.89 |
|  | *Percent change 2000 to 2017* | **-34%** | **-36%** | **-34%** | **-0.02** |
|  | **Unintentional injuries** | Males | Females | Absolute difference* | Relative difference** |
|  | 2000 | 162 | 88 | 74 | 1.84 |
|  | 2002 | 160 | 85 | 75 | 1.88 |
|  | 2004 | 158 | 83 | 75 | 1.90 |
|  | 2006 | 154 | 81 | 73 | 1.90 |
|  | 2008 | 145 | 78 | 66 | 1.86 |
|  | 2010 | 136 | 76 | 60 | 1.79 |
|  | 2012 | 129 | 74 | 55 | 1.74 |
|  | 2014 | 126 | 73 | 53 | 1.73 |
|  | 2016 | 125 | 73 | 52 | 1.71 |
|  | 2017 | 125 | 73 | 52 | 1.71 |
|  | *Percent change 2000 to 2017* | **-22%** | **-17%** | **-30%** | **-0.08** |

|  | **Denmark (cont)** |  |  |  |  |
| --- | --- | --- | --- | --- | --- |
|  | **Cardiovascular diseases** | Males | Females | Absolute difference* | Relative difference** |
|  | 2000 | 525 | 49 | 476 | 10.73 |
|  | 2002 | 537 | 64 | 474 | 8.43 |
|  | 2004 | 510 | 74 | 436 | 6.9 |
|  | 2006 | 463 | 70 | 394 | 6.66 |
|  | 2008 | 357 | 43 | 314 | 8.23 |
|  | 2010 | 277 | 20 | 257 | 13.73 |
|  | 2012 | 217 | 7 | 210 | 30.49 |
|  | 2014 | 174 | -2 | 176 | -109.59 |
|  | 2016 | 147 | -11 | 158 | -13.62 |
|  | 2017 | 139 | -15 | 154 | -9.46 |
|  | *Percent change 2000 to 2017* | **-74%** | **-130%** | **-68%** |  |

*Percent change in absolute gender differences between 2000 and 2017: 100*(male-female difference in DALY rates _2017_ - male-female difference in DALY rates _2000_ ) / (male-female differences in DALY rates _2000_). **Percent change in relative gender differences between 2000 and 2017: 1- (male/female ratio in DALY rates_2017_)/ (male/female ratio in DALY rates_2000_).

|  | **Sweden** | | | | |
| --- | --- | --- | --- | --- | --- |
|  | **All alcohol-attributed causes** | Males | Females | Absolute difference* | Relative difference** |
|  | 2000 | 1438 | 605 | 833 | 2.38 |
|  | 2002 | 1446 | 602 | 844 | 2.40 |
|  | 2004 | 1475 | 623 | 852 | 2.37 |
|  | 2006 | 1416 | 591 | 825 | 2.40 |
|  | 2008 | 1356 | 550 | 806 | 2.47 |
|  | 2010 | 1285 | 506 | 779 | 2.54 |
|  | 2012 | 1237 | 510 | 727 | 2.43 |
|  | 2014 | 1216 | 505 | 711 | 2.41 |
|  | 2016 | 1181 | 488 | 693 | 2.42 |
|  | 2017 | 1161 | 487 | 674 | 2.38 |
|  | *Percent change 2000 to 2017* | **-19%** | **-20%** | **-19%** | **0** |
|  | **Alcohol use disorder** | Males | Females | Absolute difference* | Relative difference** |
|  | 2000 | 463 | 118 | 345 | 3.61 |
|  | 2002 | 451 | 115 | 337 | 3.46 |
|  | 2004 | 451 | 115 | 337 | 3.32 |
|  | 2006 | 428 | 112 | 316 | 3.16 |
|  | 2008 | 425 | 113 | 311 | 3.11 |
|  | 2010 | 412 | 112 | 299 | 3.01 |
|  | 2012 | 384 | 112 | 272 | 2.94 |
|  | 2014 | 365 | 110 | 255 | 2.83 |
|  | 2016 | 341 | 106 | 235 | 2.76 |
|  | 2017 | 331 | 105 | 225 | 2.76 |
|  | *Percent change 2000 to 2017* | **-29%** | **-11%** | **-35%** | **0.24** |

|  | **Sweden (cont)** |  |  |  |  |
| --- | --- | --- | --- | --- | --- |
|  | **Neoplasms** | Males | Females | Absolute difference* | Relative difference** |
|  | 2000 | 235 | 270 | -35 | 0.87 |
|  | 2002 | 237 | 261 | -24 | 0.91 |
|  | 2004 | 245 | 265 | -20 | 0.92 |
|  | 2006 | 238 | 251 | -13 | 0.95 |
|  | 2008 | 234 | 239 | -6 | 0.98 |
|  | 2010 | 225 | 219 | 6 | 1.03 |
|  | 2012 | 215 | 222 | -7 | 0.97 |
|  | 2014 | 212 | 215 | -3 | 0.99 |
|  | 2016 | 207 | 205 | 2 | 1.01 |
|  | 2017 | 204 | 205 | -1 | 1.00 |
|  | *Percent change 2000 to 2017* | **-13%** | **-24%** | **-97%** | **0.87** |
|  | **Self-harm** | Males | Females | Absolute difference* | Relative difference** |
|  | 2000 | 237 | 85 | 153 | 2.79 |
|  | 2002 | 233 | 82 | 151 | 2.84 |
|  | 2004 | 234 | 88 | 146 | 2.66 |
|  | 2006 | 222 | 82 | 140 | 2.71 |
|  | 2008 | 214 | 77 | 137 | 2.78 |
|  | 2010 | 202 | 71 | 131 | 2.85 |
|  | 2012 | 198 | 73 | 125 | 2.71 |
|  | 2014 | 198 | 74 | 125 | 2.68 |
|  | 2016 | 195 | 69 | 127 | 2.83 |
|  | 2017 | 192 | 67 | 125 | 2.87 |
|  | *Percent change 2000 to 2017* | **-19%** | **-21%** | **-18%** | **-0.03** |

|  | **Sweden (cont)** |  |  |  |  |
| --- | --- | --- | --- | --- | --- |
|  | **Cirrhosis** | Males | Females | Absolute difference* | Relative difference** |
|  | 2000 | 119 | 36 | 83 | 3.31 |
|  | 2002 | 124 | 37 | 87 | 3.35 |
|  | 2004 | 128 | 38 | 91 | 3.37 |
|  | 2006 | 129 | 38 | 92 | 3.39 |
|  | 2008 | 130 | 37 | 93 | 3.51 |
|  | 2010 | 127 | 35 | 91 | 3.63 |
|  | 2012 | 121 | 37 | 84 | 3.27 |
|  | 2014 | 117 | 38 | 78 | 3.08 |
|  | 2016 | 106 | 41 | 65 | 2.59 |
|  | 2017 | 101 | 43 | 58 | 2.35 |
|  | *Percent change 2000 to 2017* | **-15%** | **19%** | **-30%** | **0.29** |
|  | **Unintentional injuries** | Males | Females | Absolute difference* | Relative difference** |
|  | 2000 | 127 | 77 | 49 | 1.65 |
|  | 2002 | 128 | 79 | 50 | 1.62 |
|  | 2004 | 131 | 81 | 50 | 1.62 |
|  | 2006 | 129 | 80 | 49 | 1.61 |
|  | 2008 | 128 | 80 | 48 | 1.60 |
|  | 2010 | 126 | 80 | 46 | 1.58 |
|  | 2012 | 126 | 80 | 46 | 1.58 |
|  | 2014 | 127 | 80 | 47 | 1.59 |
|  | 2016 | 128 | 80 | 48 | 1.60 |
|  | 2017 | 128 | 80 | 49 | 1.60 |
|  | *Percent change 2000 to 2017* | **4%** | **4%** | **0%** | **0.03** |

|  | **Sweden (cont)** |  |  |  |  |
| --- | --- | --- | --- | --- | --- |
|  | **Cardiovascular diseases** | Males | Females† | Absolute difference* | Relative difference** |
|  | 2000 | 5 | -19 | 24 | -0.3 |
|  | 2002 | 27 | -9 | 36 | -3.1 |
|  | 2004 | 42 | 0 | 43 | -173.6 |
|  | 2006 | 47 | 0 | 47 | -473.1 |
|  | 2008 | 24 | -14 | 38 | -1.7 |
|  | 2010 | 12 | -20 | 32 | -0.6 |
|  | 2012 | 17 | -19 | 37 | -0.9 |
|  | 2014 | 25 | -16 | 41 | -1.5 |
|  | 2016 | 29 | -14 | 43 | -2.1 |
|  | 2017 | 31 | -14 | 45 | -2-2 |
|  | *Percent change 2000 to 2017* | **523%** | **24%** | **88%** |  |

*Percent change in absolute gender differences between 2000 and 2017: 100*(male-female difference in DALY rates _2017_ - male-female difference in DALY rates _2000_ ) / (male-female differences in DALY rates _2000_). **Percent change in relative gender differences between 2000 and 2017: 1- male/female ratio in DALY rates_2017_ / male/female ratio in DALY rates_2000_. ‡The protective effect from alcohol on cardiovascular diseases has decreased.

|  | **Norway** | | | | |
| --- | --- | --- | --- | --- | --- |
|  | **All alcohol-attributed causes** | Males | Females | Absolute difference* | Relative difference** |
|  | 2000 | 1102 | 239 | 863 | 4.61 |
|  | 2002 | 1138 | 266 | 872 | 4.28 |
|  | 2004 | 1165 | 302 | 863 | 3.86 |
|  | 2006 | 1137 | 301 | 836 | 3.78 |
|  | 2008 | 1131 | 297 | 834 | 3.81 |
|  | 2010 | 1122 | 306 | 816 | 3.67 |
|  | 2012 | 1070 | 283 | 787 | 3.78 |
|  | 2014 | 1026 | 282 | 744 | 3.64 |
|  | 2016 | 981 | 274 | 707 | 3.58 |
|  | 2017 | 976 | 271 | 705 | 3.60 |
|  | *Percent change 2000 to 2017* | **-11%** | **13%** | **-18%** | **0.22** |
|  | **Alcohol use disorder** | Males | Females | Absolute difference* | Relative difference** |
|  | 2000 | 466 | 129 | 337 | 3.92 |
|  | 2002 | 443 | 128 | 315 | 3.92 |
|  | 2004 | 415 | 125 | 289 | 3.92 |
|  | 2006 | 395 | 125 | 270 | 3.82 |
|  | 2008 | 386 | 124 | 262 | 3.76 |
|  | 2010 | 379 | 126 | 253 | 3.68 |
|  | 2012 | 362 | 123 | 240 | 3.43 |
|  | 2014 | 348 | 123 | 225 | 3.32 |
|  | 2016 | 342 | 124 | 219 | 3.22 |
|  | 2017 | 345 | 125 | 221 | 3.15 |
|  | *Percent change 2000 to 2017* | **-26%** | **-0.3%** | **-34%** | **0.2** |

|  | **Norway (cont)** |  |  |  |  |
| --- | --- | --- | --- | --- | --- |
|  | **Neoplasms** | Males | Females | Absolute difference* | Relative differences† |
|  | 2000 | 208 | 197 | 12 | 1.06 |
|  | 2002 | 211 | 196 | 15 | 1.08 |
|  | 2004 | 209 | 192 | 17 | 1.09 |
|  | 2006 | 204 | 183 | 20 | 1.11 |
|  | 2008 | 204 | 175 | 28 | 1.17 |
|  | 2010 | 202 | 176 | 26 | 1.15 |
|  | 2012 | 193 | 165 | 28 | 1.17 |
|  | 2014 | 186 | 157 | 29 | 1.18 |
|  | 2016 | 176 | 147 | 30 | 1.20 |
|  | 2017 | 174 | 145 | 29 | 1.20 |
|  | *Percent change 2000 to 2017* | **-16%** | **-26%** | **+41%** | **-0.13** |
|  | **Self-harm** | Males | Females | Absolute difference* | Relative differences† |
|  | 2000 | 218 | 49 | 170 | 4.45 |
|  | 2002 | 213 | 50 | 163 | 4.26 |
|  | 2004 | 209 | 55 | 154 | 3.80 |
|  | 2006 | 202 | 52 | 150 | 3.88 |
|  | 2008 | 198 | 50 | 148 | 3.96 |
|  | 2010 | 197 | 50 | 147 | 3.94 |
|  | 2012 | 182 | 42 | 140 | 4.33 |
|  | 2014 | 169 | 42 | 126 | 4.02 |
|  | 2016 | 153 | 42 | 111 | 3.64 |
|  | 2017 | 153 | 42 | 112 | 3.64 |
|  | *Percent change 2000 to 2017* | **-30%** | **-14%** | **-34%** | **0.18** |

|  | **Norway (cont)** |  |  |  |  |
| --- | --- | --- | --- | --- | --- |
|  | **Cirrhosis** | Males | Females | Absolute difference* | Relative difference† |
|  | 2000 | 104 | 39 | 65 | 2.67 |
|  | 2002 | 101 | 37 | 64 | 2.73 |
|  | 2004 | 98 | 36 | 62 | 2.72 |
|  | 2006 | 92 | 34 | 57 | 2.71 |
|  | 2008 | 90 | 34 | 56 | 2.65 |
|  | 2010 | 87 | 34 | 53 | 2.56 |
|  | 2012 | 84 | 33 | 51 | 2.55 |
|  | 2014 | 82 | 32 | 51 | 2.56 |
|  | 2016 | 81 | 31 | 50 | 2.61 |
|  | 2017 | 81 | 31 | 50 | 2.61 |
|  | *Percent change 2000 to 2017* | **-20%** | **-21%** | **-23%** | **0.02** |
|  | **Unintentional Injuries** | Males | Females | Absolute difference* | Relative difference† |
|  | 2000 | 152 | 77 | 75 | 1.97 |
|  | 2002 | 154 | 79 | 75 | 1.95 |
|  | 2004 | 154 | 80 | 74 | 1.93 |
|  | 2006 | 150 | 80 | 70 | 1.88 |
|  | 2008 | 151 | 80 | 70 | 1.89 |
|  | 2010 | 151 | 81 | 70 | 1.86 |
|  | 2012 | 146 | 79 | 68 | 1.85 |
|  | 2014 | 144 | 78 | 67 | 1.85 |
|  | 2016 | 138 | 76 | 61 | 1.82 |
|  | 2017 | 137 | 75 | 61 | 1.83 |
|  | *Percent change 2000 to 2017* | **-0.1%** | **-0.3%** | **-19%** | **0.07** |

|  | **Norway (cont)** |  |  |  |  |
| --- | --- | --- | --- | --- | --- |
|  | **Cardiovascular diseases** | Males† | Females† | Absolute difference* | Relative difference† |
|  | 2000 | -272 | -216 | -55 | 1.26 |
|  | 2002 | -208 | -189 | -19 | 1.1 |
|  | 2004 | -145 | -154 | 9 | 0.94 |
|  | 2006 | -113 | -135 | 22 | 0.83 |
|  | 2008 | -99 | -124 | 25 | 0.8 |
|  | 2010 | -85 | -118 | 32 | 0.72 |
|  | 2012 | -73 | -108 | 35 | 0.67 |
|  | 2014 | -64 | -98 | 34 | 0.65 |
|  | 2016 | -60 | -95 | 35 | 0.63 |
|  | 2017 | -62 | -96 | 34 | 0.65 |
|  | *Percent change 2000 to 2017* | **-77%** | **-56%** | **-62%** |  |

*Percent change in absolute gender differences between 2000 and 2017: 100*(male-female difference in DALY rates _2017_ - male-female difference in DALY rates _2000_ ) / (male-female differences in DALY rates _2000_). **Percent change in relative gender differences between 2000 and 2017: 1- male/female ratio in DALY rates_2017_ / male/female ratio in DALY rates_2000_. †The protective effect from alcohol on cardiovascular diseases has decreased.
